# Red-Osier Dogwood Extracts Prevent Inflammatory Responses in Caco-2 Cells and a Caco-2 BBe1/EA.hy926 Cell Co-Culture Model

**DOI:** 10.3390/antiox8100428

**Published:** 2019-09-25

**Authors:** Qian Jiang, Hua Zhang, Runqiang Yang, Qianru Hui, Yuhuan Chen, Lili Mats, Rong Tsao, Chengbo Yang

**Affiliations:** 1 Department of Animal Science, University of Manitoba, Winnipeg, MB R3T 2N2, Canada; qian.jiang@umanitoba.ca (Q.J.); yangrq@njau.edu.cn (R.Y.); huiq@myumanitoba.ca (Q.H.); 2 Laboratory of Animal Nutritional Physiology and Metabolic Process, Institute of Subtropical Agriculture Chinese Academy of Sciences, Changsha 410125, China; 3 Food Nutrition and Safety, Department of Pharmacy, Jiangxi University of Traditional Chinese Medicine, Nanchang 330004, China; 4 Guelph Research & Development Centre, Agriculture and Agri-Food Canada, Guelph, ON N1G 5C9, Canadalili.mats@canada.ca (L.M.); Rong.Cao@canada.ca (R.T.); 5 State Key Laboratory of Food Science and Technology, University of Nanchang, Nanchang 330047, China

**Keywords:** red-osier dogwood, polyphenols, antioxidants, TNF-α, ox-LDL, Caco-2 BBe-1/EA.hy926 co-culture cell model

## Abstract

Red-osier dogwood extracts (RDE) contain high levels of phenolic compounds which have been recognized as natural antioxidants. In this study, the potential of RDE to prevent cardiovascular diseases (CVDs) was evaluated using Caco-2 cells and a co-culture model of Caco-2 BBe1/EA.hy926 cells in Transwell^®^ plates. The results showed that RDE supplementation significantly prevented interleukin-8 (IL-8) production and suppressed the gene expression of IL-8, tumor necrosis factor-alpha (TNF-α), interleukin-6 (IL-6), intercellular adhesion molecule-1 (ICAM-1), vascular cell adhesion molecule-1 (VCAM-1), and cyclooxygenase 2 (COX-2) in the TNF-α inflamed Caco-2 cells. Meanwhile, the polyphenols (quercetin-3-glucoside, quercetin-glucuronide, rutin, quercetin-3-*O*-malonylglucoside, and kaempferol-glucoside) in the RDE were validated to be absorbed by Caco-2 BBe1 cells and transported to the basal chamber where EA.hy926 cells were located during 12 h incubation. The transported polyphenols were able to prevent IL-8 production and suppress the gene expression of proinflammatory mediators (TNF-α, ICAM-1, VCAM-1, and COX-2) in the TNF-α or oxidized low-density lipoprotein (ox-LDL) treated EA.hy926 cells. These novel findings demonstrated that phenolic compounds in RDE can be transported to the cardiovascular system by intestinal absorption and mitigate the inflammatory responses of vascular endothelial cells, indicating that RDE could be a natural resource of polyphenols to prevent inflammation cytokine or oxidized lipid-induced CVDs.

## 1. Introduction 

Immune system disorder and redox imbalance in the gut may cause chronic intestinal inflammation and defective barrier functions [1]. This subsequently increases intestinal permeability and bacterial endotoxin accumulation in the circulatory system, which results in cardiovascular inflammation and related diseases [2]. Cardiovascular diseases (CVDs) remain a leading cause of death globally, supported by clinical and epidemiologic evidence showing that around 17.9 million people died from CVDs in 2016 [3]. The ongoing inflammatory events induce irreversible and permanent damage in arteries, by which the progressive development of CVDs, including heart attack and stroke, is triggered [4]. Current findings have elucidated that the increased release of proinflammatory mediators including interleukin-8 (IL-8), tumor necrosis factor-alpha (TNF-α), interleukin-6 (IL-6), and vascular cell adhesion molecule 1 (VCAM-1), as well as the anti-inflammatory cytokine interleukin-10 (IL-10), are involved in the recruitments of monocytes or macrophages to endothelial cells (ECs) and pathogenesis of CVDs [5,6]. Therefore, preventing chronic inflammation by reducing the release of proinflammatory mediators could be an effective way to prevent intestinal and CVDs.

Red-osier dogwood (*Cornus sericea L. ssp. sericea*), the most widespread native species of North America, has been reported to improve animal growth performance and feed digestibility [7]. Polyphenols are natural chemicals in plants and have been widely reported to benefit health [8]. The red-osier dogwood harvested near Swan River, Manitoba, contains high levels of phenolic compounds, in particular, rutin and quercetin, both of which have exhibited anti-inflammatory and antioxidative features [9,10,11]. In order to play anti-inflammatory and antioxidative roles in the cardiovascular system, phenolic compounds in the red-osier dogwood extract (RDE) must be absorbed and transported to the blood circulation by the intestinal epithelial cells [12,13]. In this study, to mimic the absorption of phenolic compounds by the intestine, the co-culture model of Caco-2 BBe1 [14] (a human cell line for predicting intestinal barrier and absorption functions)/EA.hy926 cells [15] (an established endothelial cell line from human umbilical vein) was used, and the absorption and transport of polyphenols by Caco-2 BBe1 cells in the co-culture model were analyzed. Thereafter, the anti-inflammatory effects of transported polyphenols on TNF-α or ox-LDL treated EA.hy926 cells in the co-culture model of Caco-2 BBe1/EA.hy926 were determined. To the best of our knowledge, this study is the first in vitro study evaluating the absorption of phenolic compounds in RDE, as well as their potential for preventing CVDs.

## 2. Materials and Methods 

### 2.1. Materials and Reagents

RDE powder (harvested, extracted, and spray-dried from leaves; total phenolic content = 26.51%) was provided by Red Dog Enterprises Ltd. (Swan River, Manitoba, Canada). Hank’s balanced salt solution (HBSS), DMEM/F12 medium, trypsin (0.25%), penicillin, streptomycin, sodium pyruvate with non-essential amino acid and amphotericin B, and fetal bovine serum (FBS) were purchased from Thermo Fisher Scientific (Mississauga, Ontario, Canada). T-25 and T-75 culture flasks, Transwell^®^ plates (0.4 μm pore size) and cell culture plates were purchased from Corning, Fisher Scientific (Ottawa, Ontario, Canada). Caco-2, Caco-2 BBe1, and EA.hy926 cells were purchased from the American Type Culture Collection (Manassas, Virginia, United States).

### 2.2. Maintenance of Monolayer Caco-2 Cells

Caco-2 cells between the 50th to 60th passages were seeded on the apical compartment of Transwell^®^ plates at a cell density of 3 × 10^4^ cells/well and were cultured in a DMEM high glucose medium supplemented with 10% FBS, 50 U/mL penicillin-streptomycin, 25 mM 4-(2-hydroxyethyl) piperazine-1-ethanesulfonic acid, 1 mM sodium pyruvate with non-essential amino acid, and 0.01 µg/mL human transferrin at 37 °C in a gas and humidity controlled 5% CO_2_ cell culture incubator (Thermo Fisher Scientific, Waltham, Massachusetts, United States). The culture medium was changed every other day until the cell’s confluency and polarization was reached. Cell monolayers that displayed a transepithelial electrical resistance (TEER) greater than 400 Ω∙cm^2^ on the 9th day were incorporated into actual experiments. The TEER values of the monolayers were determined after equilibrating the cells with HBSS for 30 min at 37 °C. A Millicell-ERS Volt-Ohm Meter (Millipore, Bedford, Massachusetts, United States) was used for the TEER determination. 

### 2.3. Co-Culture Model of Caco-2 BBe1/EA.hy926 Cells

The Caco-2 BBe1 cells were seeded on the apical compartment of the Transwell^®^ plates at a density of 3 × 10^4^ cells/well. The culture medium was changed every other day before the cells reached confluency, and then changed every day once the cells reached 100% confluency. The EA.hy926 cells were seeded on the basolateral compartment of the Transwell^®^ plate when Caco-2 BBe1 monolayers on the apical compartment displayed TEER values greater than 700 Ω∙cm^2^ on the 18th day (post-seeding). 

### 2.4. Determination of RDE Cytotoxicity on Caco-2 Cells and Caco-2 BBe1 Cells

To apply an optimal concentrations of RDE in our study, the cytotoxicity of RDE on Caco-2 cells and Caco-2 BBe1 cells was investigated by treating the cells (preseeded on the 96-well cell culture plates) with a series of concentrations (0, 3.125, 6.25, 12.5, 50, 100, and 200 μg/mL) of RDE for 24 h. Water-soluble tetrazolium salts (WST-1, Sigma Aldrich, Saint Louis, Missouri, United States) were used to determine the relative cell viability of the treated cells. The experimental procedures were described in our previous study [16]. 

### 2.5. Anti-Inflammatory Experiments on Monolayer Caco-2 Cells

Caco-2 cells were pretreated with 50 μg/mL of RDE for 1 h. Next, the tumor necrosis factor-α (TNF-α) was added to the apical compartment of a Transwell® plate at a final concentration of 2 ng/mL and incubated with cells for 4 h. The supernatants from the negative control cells (untreated), positive control cells (treated with TNF-α alone), and treatment cells (RDE pretreated and then TNF-α treated) were collected and stored at −80 °C for measuring interleukin-8 (IL-8) release using enzyme-linked immunosorbent assay (ELISA). Caco-2 cells treated with 50 μg/mL RDE were washed twice with ice-cold phosphate buffer solution (PBS, pH 7.0) and then stored at −80 °C for real-time polymerase chain reaction (PCR).

### 2.6. Anti-Inflammatory Experiments on EA.hy926 Cells in the Caco-2 BBe1/ EA.hy926 Cells Co-Culture Model

A final concentration of 100 μg/mL RDE was added to the apical compartment in which Caco-2 BBe1 cells were grown to form a barrier. After 1 h preincubation, co-cultured EA.hy926 cells were stimulated with TNF-α (2 ng/mL) in the basolateral compartment for a 4 h incubation. Supernatants of the basolateral compartment were sampled for IL-8 secretion measurement, and the cells were collected for real-time PCR. To investigate the effects of RDE on ox-LDL induced inflammatory responses, RDE was added into the apical compartment at a final concentration of 100 μg/mL in a co-culture model. After 1 h preincubation, co-cultured EA.hy 926 cells were stimulated with ox-LDL (100 μg/mL) in the basolateral compartment for a 6 h incubation. Supernatants of the basolateral compartment were sampled for IL-8 secretion measurement, and the cells were collected for real-time PCR.

### 2.7. Absorption and Transepithelial Transport Experiments

The experiments were conducted according to the procedures which were described in a previous study [17]. After washing the polarized and differentiated Caco-2 BBe1 with prewarmed HBSS, the RDE (100 μg/mL equilibrated into 0.5 mL of HBSS, pH 6.4 for 30 min at 37 °C) was applied to the apical compartment of each well and incubated with the Caco-2 BBe1 cells for up to 24 h. One mL of HBSS solution (containing 10 mM HEPES, pH 7.4) was added to the basolateral compartment. The transepithelial transport of phenolics was subsequently assessed at 2, 4, 8, 12, and 24 h intervals in order to determine the metabolic kinetics of phenolics. An equal volume of methanol (containing 2% formic acid) was added to the collected solution(s) obtained from the basolateral compartment. This mixture was then applied to Strata^TM^-X polymeric solid phase extraction (SPE) cartridges (30 mg, Phenomenex, Torrance, California, United States) and later evaporated to dryness for subsequent HPLC (high performance liquid chromatography) analysis (described below). After removing culture medium from each individual insert, 500 µL of cold PBS was added to each insert before extracting and tracking the cellular uptake of phenolics. In brief, the detached cells were transferred to 1.5 mL tube(s) and homogenized for 1 min by brief sonication (Qsonica Sonicator Q500, Fisher Scientific, Canada). Short centrifugation at 2000× *g* for 5 min with the addition of 500 µL methanol (2% formic acid) was conducted in order to completely extract intracellular phenolics. The supernatant was collected and cleaned up before proceeding to the HPLC analysis. 

Phenolics transported by intestinal epithelial cells were analyzed using an Agilent 1100 series HPLC system, as previously described [17]. The phenolics were separated on a Phenomenex Luna phenyl-hexyl column (5 m, 250 × 4.6 mm) (Phenomenex Inc., Torrance, California, United States). A binary mobile phase consisting of 5% formic acid in water (v/v) (solvent A) and 95% methanol mixed with 5% acetonitrile (v/v) (solvent B) was used. The solvent gradient was as follows: 0−20 min, 0%–60% B; 20–25 min, 60%–100% B; 25–27 min, 100% B; and 27–33.5 min, 100%–0% B. Peaks were monitored at 360 nm and 520 nm, respectively. Subsequently, LC-MS/MS analysis was performed using a Thermo® Scientific Q-Exactive™ Orbitrap mass spectrometer equipped with a Vanquish™ Flex Binary UPLC System (Waltham, MA, USA). Data were acquired using Thermo Scientific™ Xcalibur™ 4.2 software and Thermo Scientific™ Standard Integration Software (SII). The chromatographic separation was performed on a Kinetex XB-C18 100A HPLC column (100 × 4.6 mm, 2.6 µm, Phenomenex Inc., Torrance, California, United States). The binary mobile phase consisted of solvent A (99.9% H2O/ 0.1% formic acid) and solvent B (94.9% MeOH/ 5% ACN/ 0.1% formic acid). Negative ionization mode was used; the spray voltage was set at 2.8 kV. Mass spectrometry data were collected using the Full-MS/DDMS2 (TopN = 10) method, with NCE set at 30 and the intensity threshold set at 1.0 × 10^5^ counts. Thermo FreeStyle™ 1.5 software was used for data analysis. 

The transported efficiency was then calculated as the ratio of the obtained phenolic peak area in the basolateral compartment to that in the apical compartment. The quantification of identified polyphenols was performed through external standards of quercetin-3-glucoside using a linear curve established with solutions containing predefined concentrations of each flavonoid. Data analysis was then conducted using the provided Agilent ChemStation software (Santa Clara, California, United States). 

### 2.8. Interleukin-8 (IL-8) Measurements 

The concentration of IL-8 produced by mono-cultured Caco-2 or EA.hy926 cells in the Caco-2 BBe1/EA.hy926 co-culture model was determined using ELISA assay kits (Invitrogen, Carlsbad, California, United States). The procedures of the measurement were carried out according to the manufacturer’s instructions. In general, 100 μL of the supernatant of culture medium was used for IL-8 assays. After the reactions for 30 min, the microplates were read at 450 nm using a Synergy™ H4 Hybrid Multi-Mode Microplate Reader (BioTek, Winooski, Vermont, United States). Final concentrations of IL-8 were calculated from a calibration curve, and the IL-8 levels were presented as pg/mL. 

### 2.9. RNA Extraction and Real-Time PCR

Total RNA was extracted from cells using Trizol reagent (Invitrogen) following the manufacturer’s protocol. One microgram of total RNA was reversely transcribed into cDNA using the iScript^TM^ cDNA Synthesis kit (Bio-Rad, Hercules, California, United States) following the manufacturer’s instruction. Quantitative real-time PCR was performed using SYBR Green Supermix (Bio-Rad) on a CFX Connect™ Real-Time PCR Detection System (Bio-Rad). The reference gene was GAPDH. The primers for real-time PCR analysis were designed with Primer-Blast based on the published cDNA sequences in the Gene Bank (https://www.ncbi.nlm.nih.gov/tools/primer-blast/). Information regarding the detected genes and primers is shown in Table 1. 

### 2.10. Statistical Analysis

Data were presented as means ± standard error of the means (SEM). The statistical analyses were performed with the GraphPad Prism 8.01 software (San Diego, California, United States). Differences between the treatments were evaluated by one-way ANOVA. *P*-values less than 0.05 (*p* < 0.05) were considered statistically significant.

## 3. Results 

### 3.1. Cytotoxicity of RDE on Caco-2 and Caco-2 BBe1 Cells

**Figure 1:**
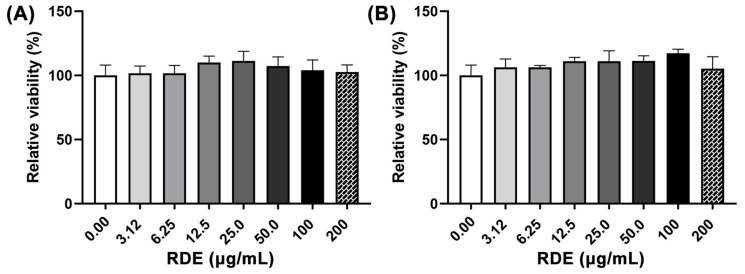
Dose-effects of red-osier dogwood extracts (RDE) on the viability of Caco-2 (**A**) and Caco-2 BBe1 cells (**B**). Cells were seeded into a 96-well plate and cultured until 90% confluence was reached, and then treated with different concentrations of RDE for 24 h. Cell viability was measured using a WST-1 kit. Values are presented as means ± SEM, n = 4.

The effects of different concentrations (0, 3.125, 6.25, 12.5, 50, 100, and 200 μg/mL) of RDE on cell viability of Caco-2 cells and Caco-2 BBe1 cells are shown in Figure 1. No significant difference (*p* > 0.05) of cell viability among these groups was observed. 

### 3.2. Dose-Effect of RDE on the IL-8 Production in Monolayer Caco-2 Cells

**Figure 2:**
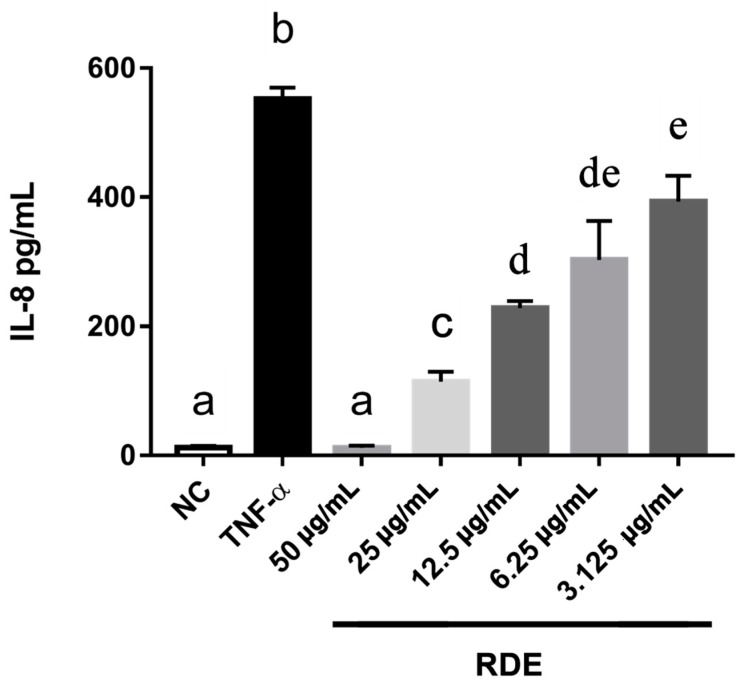
Production of IL-8 in Caco-2 cells in response to no inflammation (NC), TNF-α induced inflammation (TNF-α), or a series of RDE concentrations with the following TNF-α induced inflammation. Caco-2 cells were pretreated with a series of concentrations (3.125, 6.25, 12.5, 25, and 50 μg/mL) of RDE for 1 h, followed by incubation with 2 ng/mL TNF-α for 4 h. Values are presented as means ± SEM, n = 4. Shared letters indicate no significant difference (*p* > 0.05).

To investigate the anti-inflammatory effects of RDE on the intestinal epithelial cells, we determined the IL-8 production in the monolayer Caco-2 cells using ELISA. Results showed that the proinflammatory mediator IL-8 was produced significantly (*p* < 0.05) higher by the TNF-α inflamed Caco-2 cells as compared with that by the untreated cells. One h pretreatment with RDE (final concentrations of 3.125, 6.25, 12.5, 25.0, and 50.0 μg/mL) significantly (*p* < 0.05) reduced the IL-8 production in monolayer Caco-2 cells in a concentration-dependent manner, as shown in Figure 2.

### 3.3. Effects of RDE on the Cytokine Gene Expression in the Monolayer Caco-2 Cells

**Figure 3:**
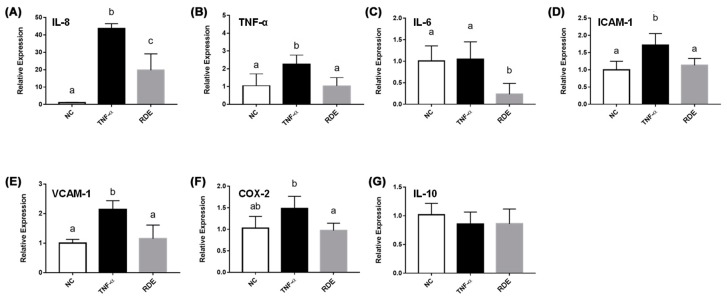
Gene expression of inflammatory mediators in no treatment (NC), TNF-α treated (TNF-α), or RDE then TNF-α treated (RDE) Caco-2 cells. Caco-2 cells were pretreated with 50 μg/mL of RDE for 1 h, followed by 2 ng/mL TNF-α incubation for another 4 h. The relative mRNA expression was measured by real-time PCR. Gene expressions of (**A**) IL-8, (**B**) TNF-α, (**C**) IL-6, (**D**) ICAM-1, (**E**) VCAM-1, (**F**) COX-2, and (**G**) IL-10 are shown. Values are presented as means ± SEM, n = 4. Shared letters indicate no significant difference (*p* > 0.05).

As shown in Figure 3, the TNF-α treatment enhanced (*p* < 0.05) the gene expressions of IL-8, TNF-α, IL-6, ICAM-1, and VCAM-1 in the monolayer Caco-2 cells. Pretreatment with 50.0 μg/mL RDE for 1 h significantly (*p* < 0.05) suppressed the expressions of these genes. The gene expression of IL-10 in the Caco-2 cells was not affected (*p* > 0.05) by the TNF-α or RDE treatments.

### 3.4. Absorption and Transport of Polyphenols by Caco-2 BBe1 Cells 

**Figure 4:**
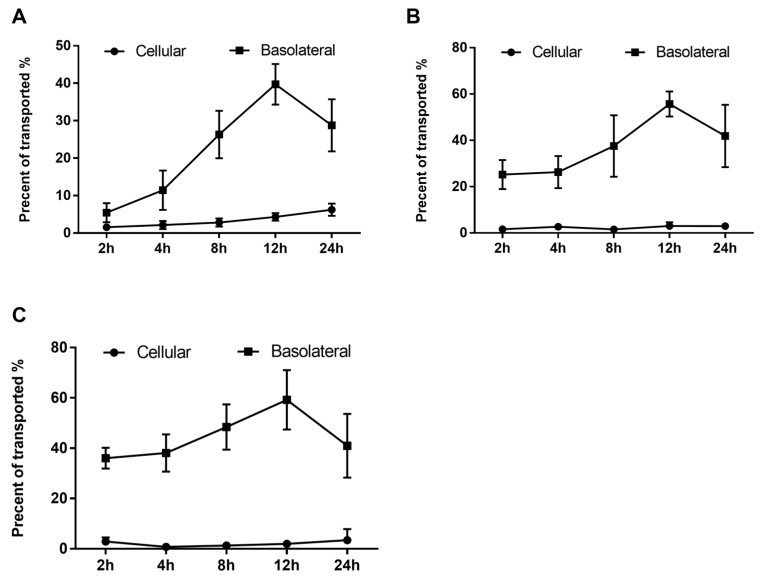
Uptake and transport of RDE-derived phenolics by Caco-2 BBe1 monolayers. After incubating the Caco-2 BBe1 cells on the apical compartment with 100 μg/mL RDE for 2, 4, 8, 12, and 24 h, three main peaks were then identified by HPLC to indicate the absorbed and transported phenolics. The permeability efficiency of peak 1 (**A**), peak 2 (**B**), and peak 3 (**C**) were then assessed by HPLC analysis. The phenolics content in the Caco-2 BBe1cell lysate or the culture medium of the basolateral compartment were calculated and normalized by phenolics in apical compartment throughout the incubation period. Phenolics uptake by Caco-2 BBe1 cells is shown in filled circles, and the transported phenolics in the basolateral compartment are shown in squares. Values are presented as means ± SEM, n = 5.

The transepithelial transport of phenolics was assessed at 2, 4, 8, 12, and 24 h intervals in order to characterize and determine the actual concentration of transported phenolics and their effect on the EA.hy926 cells. As shown in Figure 4, the absorbed or transported phenolics were accumulated throughout the incubation period. In addition, the highest transport efficiency of all three peaks was detected at 12 h of incubation, and after that, the transported content started to decrease (Figure 4). 

**Figure 5:**
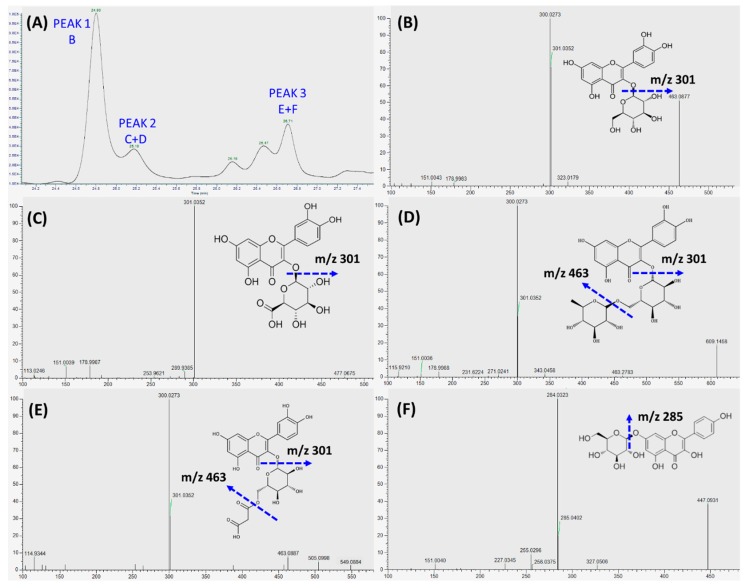
Identification of transepithelial phenolic compounds within 24 h RDE incubation. RDE (100 µg/mL) was supplemented to the apical compartment (Caco-2 BBe1 cell monolayer located) of Transwell® plate. (**A**) Chromatographic profiles (280 nm) of the absorbed and transported phenolic compounds in the medium collected from the basolateral compartment. The fragmentation pattern of the major identified polyphenols from three peaks were characterized by LC-MS/MS, the identified flavonoid for peak 1 are shown in (**B**), the identified polyphenols for peak 2 are shown in (**C**) and (**D**), and the identified polyphenols for peak 3 are shown in (**E**) and (**F**).

As shown in Figure 5A, the major phenolics collected in the basolateral compartment of Transwell^®^ plate were separated by LC into peak 1, 2, and 3, and, subsequently, characterized by LC-MS/MS. Quercetin-3-glucoside was identified as the sole flavonoid in peak 1 (Figure 5B), whereas a mixture of polyphenols, i.e., quercetin-glucuronide (Figure 5C) and rutin (Figure 5D) was identified in peak 2, and quercetin-3-*O*-malonylglucoside (Figure 5E) and kaempferol-glucoside (Figure 5F) were identified in peak 3. Quercetin-3-glucoside (Figure 5B) was identified as the main transported flavonoid, which can reach a peak level of 8.49 ± 0.88 μg/mL within 12 h incubation. 

### 3.5. Effects of Transported Polyphenols on the IL-8 Secretion and Cytokine Gene Expression in the TNF-α Inflamed EA.hy962 Cells 

**Figure 6:**
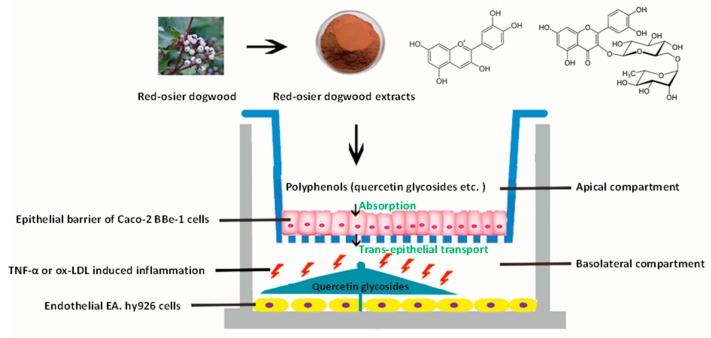
Graphical scheme of the co-culture model of a Caco-2 BBe1/EA.hy926 cell used for the potential evaluation of RDE for preventing CVDs. A final concentration of 100 μg/mL RDE was added to the apical compartment in which Caco-2 BBe1 cells were grown to form a barrier. After 1 h preincubation, co-cultured EA.hy926 cells were stimulated with TNF-α (2 ng/mL) or ox-LDL (100 μg/mL) in the basolateral compartment for 4 h and 6 h, respectively. The transported polyphenols were identified and quantified by LC-MS/MS method. The interleukin-8 (IL-8) production and gene expressions of IL-8, tumor necrosis factor-alpha (TNF-α), interleukin-6 (IL-6), intercellular adhesion molecule-1 (ICAM-1), vascular cell adhesion molecule-1 (VCAM-1), cyclooxygenase 2 (COX-2), and interleukin-10 (IL-10) of the EA.hy926 cells were determined for the purpose of evaluation.

The graphical scheme of the co-culture model of a Caco-2 BBe1/EA.hy926 cell used for the potential evaluation of RDE for preventing CVDs is shown in Figure 6. 

**Figure 7:**
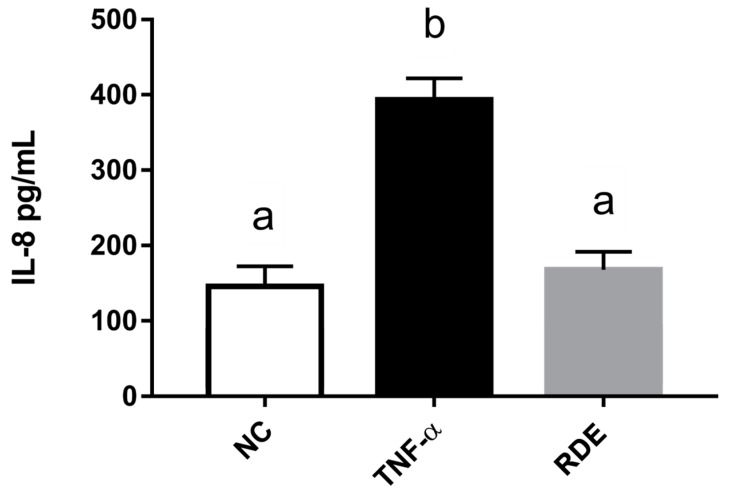
Production of IL-8 by EA.hy926 cells in the Caco-2 BBe1/ EA.hy926 co-culture model in response to no inflammation (NC), TNF-α induced inflammation (TNF-α), or RDE pretreatment with the following TNF-α induced inflammation (RDE). Caco-2 BBe1 cells on the apical compartment were supplemented with 100 μg/mL RDE for 1 h, followed by a 2 ng/mL incubation of TNF-α on the EA.hy926 cells for 4 h. Values are presented as means ± SEM, n = 4. Shared letters indicate no significant difference (*p* > 0.05).

To investigate the anti-inflammatory effects of RDE on endothelial cells, we determined the IL-8 concentrations in the basolateral compartment (EA.hy926 cells located) using ELISA. Results showed that the TNF-α treatment significantly (*p* < 0.05) increased the IL-8 production of EA.hy926 cells as compared with the untreated cells. RDE supplemented to the apical compartment (Caco-2 BBe1 located) significantly (*p* < 0.05) reduced IL-8 production in TNF-α inflamed EA.hy926 cells, as shown in Figure 7. 

**Figure 8:**
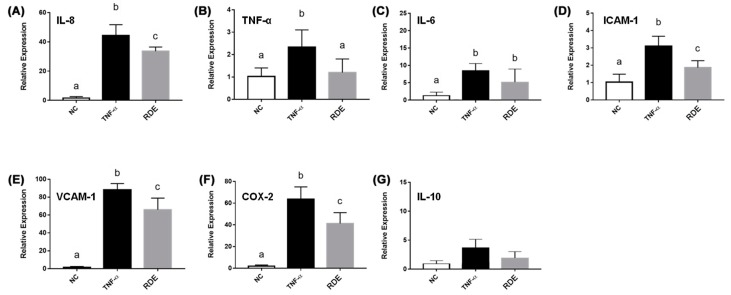
Gene expression of inflammatory mediators in no treatment (NC), TNF-α treated (TNF-α), or RDE then TNF-α treated (RDE) EA.hy926 cells in the Caco-2 BBe1/EA.hy926 co-culture model. Caco-2 BBe1 cells on the apical compartment were supplemented with 100 μg/mL RDE for 1 h, followed by a 2 ng/mL incubation of TNF-α on the EA.hy926 cells for 4 h. The gene expressions of (**A**) IL-8, (**B**) TNF-α, (**C**) IL-6, (**D**) ICAM-1, (**E**) VCAM-1, (**F**) COX-2, and (**G**) IL-10 are shown. Values are presented as means ± SEM, n = 4. Shared letters indicate no significant difference (*p* > 0.05).

The TNF-α treatment enhanced (*p* < 0.05) the gene expressions of IL-8, TNF-α, IL-6, ICAM-1, VCAM-1, and COX-2 in the EA.hy962 cells (Figure 8). The RDE supplementation at 100 μg/mL to the apical compartment for 1 h significantly (*p* < 0.05) suppressed the expressions of IL-8, TNF-α, ICAM-1, VCAM-1, and COX-2 in the TNF-α inflamed EA.hy962 cells. Gene expressions of IL-6 and IL-10 in the TNF-α inflamed EA.hy962 cells were not attenuated (*p* > 0.05) by this RDE supplementation.

### 3.6. Effects of Transported Polyphenols on the IL-8 Secretion and Cytokine Gene Expression in the Ox-LDL Inflamed EA.hy962 Cells 

**Figure 9:**
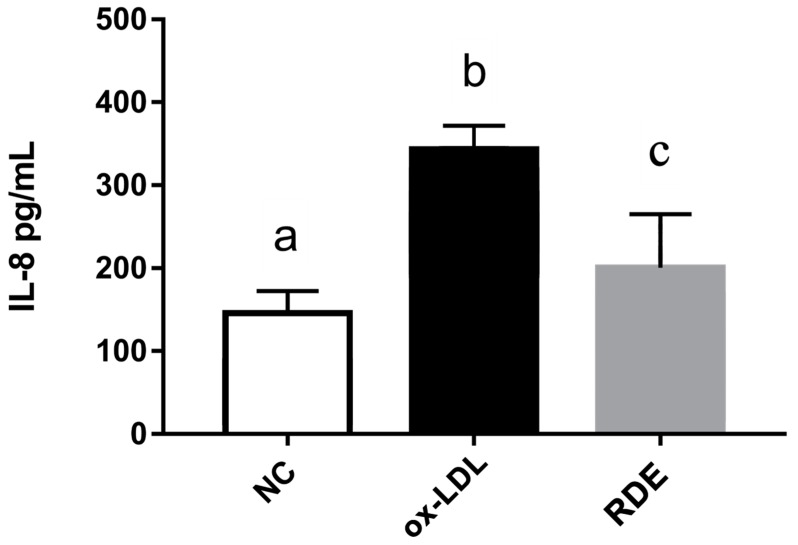
IL-8 production by EA.hy926 cells in the Caco-2 BBe1/ EA.hy926 co-culture model in response to no treatment (NC), ox-LDL induced inflammation (ox-LDL), or RDE treatment with a following ox-LDL induced inflammation (RDE). Caco-2 BBe1 cells on the apical compartment were supplemented with 100 μg/mL RDE for 1 h, followed by an incubation of 100 μg/mL ox-LDL on the EA.hy926 cells for 6 h. Values are presented as means ± SEM, n = 4. Shared letters indicate no significant difference (*p* > 0.05).

**Figure 10:**
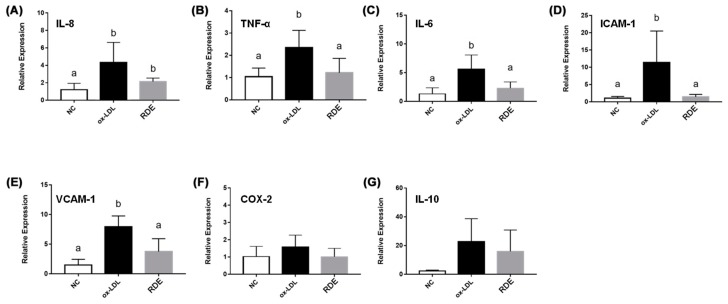
Gene expression of inflammatory mediators in no treatment (NC), ox-LDL treated (ox-LDL), or RDE then ox-LDL treated EA.hy926 cells in the Caco-2 BBe1/EA.hy926 co-culture model. Caco-2 BBe1 cells on the apical compartment were supplemented with 100 μg/mL RDE for 1 h, followed by an incubation of 100 μg/mL ox-LDL on the EA.hy926 cells for 6 h. The gene expression of (**A**) IL-8, (**B**) TNF-α, (**C**) IL-6, (**D**) ICAM-1, (**E**) VCAM-1, (**F**) COX-2, and (**G**) IL-10 are shown. Values are presented as means ± SEM, n = 4. Shared letters indicate no significant difference (*p* > 0.05).

Results show that the ox-LDL treatment significantly (*p* < 0.05) increased the IL-8 production of EA.hy926 cells as compared with untreated cells. The RDE supplemented to the apical compartment (Caco-2 BBe1 located) significantly (*p* < 0.05) reduced IL-8 production in ox-LDL inflamed EA.hy926 cells (Figure 9). The ox-LDL treatment enhanced (*p* < 0.05) the gene expressions of IL-9, TNF-α, IL-6, ICAM-1, and VCAM-1 in the EA.hy962 cells (Figure 10). The RDE supplemented at 100 μg/mL to the apical compartment for 1 h significantly (*p* < 0.05) suppressed the expressions of TNF-α, ICAM-1, and VCAM-1 in the ox-LDL inflamed EA.hy962 cells. Gene expressions of IL-8, COX-2, and IL-10 in the ox-LDL inflamed EA.hy962 cells were not attenuated (*p* > 0.05) by this RDE supplementation.

## 4. Discussion 

Cardiovascular diseases (CVDs) remain a leading cause of death globally and have been correlated with inflammatory responses in the gut-blood-barrier and irreversible inflammatory damage in the blood vessel endothelium [18,19]. Chronic inflammation in intestinal epithelial cells could lead to a leaky gut-blood-barrier and result in increased intestinal permeability to bacterial endotoxin [20,21]. Luminal endothelial cells in the blood vessels produce cytokines (e.g., TNF-α), chemokines (e.g., IL-8 and MCP-1), and adhesion molecules (e.g., ICAM-1) in response to endotoxin-induced inflammation, during which CVDs are developed [22]. Thus, the anti-inflammatory strategy is an acknowledged method for preventing CVDs. Results in this study strongly confirmed the anti-inflammatory effects of RDE on both intestinal epithelial cells and vascular endothelial cells, which point to its potential in reducing the risk associated with CVDs. 

RDE at concentrations below 200 μg/mL was not toxic to either Caco-2 or Caco-2 BBe1 cells, and thus the concentration of 50 μg/mL was applied for the anti-inflammatory experiments on Caco-2, and 100 μg/mL on the Caco-2 BBe1/EA.hy926 cell co-culture model. First, we investigated the anti-inflammatory effects of RDE on intestinal epithelial cells by using the monolayer Caco-2 cell model, as the RDE could directly affect intestinal epithelial cells before getting through the blood intestinal barrier. Similar to our previous study showing that the RDE strengthens intestinal barrier function in the H_2_O_2_ treated Caco-2 cells [23], here, we revealed that the RDE preincubation reduced IL-8 secretion and downregulated the gene expressions of IL-8, TNF-α, IL-6, ICAM-1, and VCAM-1 in TNF-α inflamed Caco-2 cells, confirming the role of RDE both in alleviating the oxidative damage and inflammatory response of the gut-blood-barrier. It is noteworthy that gene expression of IL-6 in the TNF-α inflamed Caco-2 cells was extremely downregulated by 50 μg/mL of RDE supplementation (Figure 3C). However, the IL-10 expression in the Caco-2 cells was not affected by the TNF-α stimulation or the RDE treatment (Figure 3G). Although the molecular mechanism cannot be well clarified based on the current results, we propose that IL-6 associated pathway, rather than IL-10 activation, might be involved in the molecular mechanism of RDE in preventing the IL-8 production and inflammation in Caco-2 cells. 

To protect the vascular endothelial tissue from inflammation-induced damage, the active ingredients in the RDE must be absorbed by the gut and transported to the blood circulation without any loss in biological activity. One possible factor that may affect the biological activity of the active compounds in RDE is transportation through the intestinal epithelium [24,25]. To mimic the in vivo transport of RDE bioactives by the intestinal epithelium, an in vitro co-culture model consisting of Caco-2 BBe1 cells and endothelial EA.hy926 cells in the Transwell^®^ plate was used. The results from the transport experiment (Figure 4; Figure 5) showed that predominant active ingredients (quercetin-3-glucoside, quercetin-glucuronide, rutin, quercetin-3-*O*-malonylglucoside, and kaempferol-glucoside) in RDE were rapidly absorbed and transported by the Caco-2 BBe1 cells to the basolateral compartment. These observations suggest that RDE, when used as a dietary supplement, could be effectively delivered into the blood circulation and can potentially have direct effects on the cardiovascular system. Consistent with a previous study showing polyphenols gradually degrades under a cell culture condition [26], all the detected polyphenols in this study showed a decreasing transport efficacy during the 12 to 24 h incubation which could be attributed to a prolonged incubation-induced degradation of polyphenols. As shown in our transport experiment, the main transported flavonoid from the 100 μg/mL RDE incubation is quercetin-3-glucoside, which can reach a peak level of 8.49 ± 0.88 μg/mL within 12 h incubation. As indicated by one previous study [27], the threshold safe concentration of quercetin-3-glucoside to EA.hy926 cell line should be higher than 80 μmol/L, that is, 24 μg/mL. Therefore, we proposed that 100 μg/mL of RDE applied to the Caco-2 BBe1 is also a safe concentration for EA.hy926 cells located in the basolateral chamber.

TNF-α is one of the most important inflammatory mediators in endothelial cells [28], and a large number of studies suggest a link between TNF-α mediated inflammation damage and endothelial dysfunction [29]. Our study showed 2 ng/mL TNF-α significantly enhanced the IL-8 production and proinflammation related gene expressions (IL-8, TNF-α, IL-6, ICAM-1, VCAM-1, and COX-2) in EA.hy926 cells, indicating that the inflammatory responses were triggered by this TNF-α stimulation. These results are consistent with one previous study [30], in which 1 ng/mL TNF-α induced secretions of IL-8, monocyte chemoattractant protein-1 (MCP-1), vascular endothelial growth factor (VEGF), and ICAM-1 in EA.hy926 cells. Clinical evidence has shown that disorders of lipid metabolism are associated with the pathogenesis of atherosclerosis [31,32]. An oxidized form of LDL (ox-LDL) has been reported to disturb redox balance and trigger the NF-κB mediated inflammation [33,34]. Correlations between circulating ox-LDL and CVD development has been observed in various studies [35]. Our study showed that 100 ng/mL ox-LDL significantly enhanced IL-8 production and proinflammatory gene expressions (e.g., IL-8, TNF-α, IL-6, ICAM-1, and VCAM-1) in EA.hy926 cells, indicating that the oxidized lipid-induced CVD model was successfully established. In this study, RDE supplemented to the apical compartment (Caco-2 BBe1 cells located) significantly downregulated the gene expressions of proinflammation associated markers (TNF-α, ICAM-1, VCAM-1, and COX-2) in EA.hy926 cells treated by TNF-α or ox-LDL, indicating the strong anti-inflammatory role of RDE. Interestingly, the gene expression of IL-10 in the EA.hy926 cells was numerically upregulated (*P* > 0.05) by both TNF-α (Figure 8G) and ox-LDL (Figure 10G) stimulation, indicating an activation of IL-10 mediated anti-inflammatory pathway in the EA.hy926 cells. However, the gene expression of IL-10 in Caco-2 cells was not affected by the TNF-α stimulation (Figure 3G), indicating a different pattern of IL-10 gene activation between Caco-2 cells and EA.hy926 cells, and this difference might be attributed to the different cell types and physiological function. In addition, the present results revealed different mechanisms by which the factors TNF-α and ox-LDL stimulated inflammation in EA.hy926 cells. To be specific, the TNF-α supplemented to EA.hy926 cells extremely upregulated the gene expressions of VCAM-1 and COX-2 by more than 80-fold and was accompanied with a slight upregulation of ICAM-1, however, the ox-LDL supplemented to EA.hy926 cells slightly upregulated gene expressions of VCAM-1 and COX-2 and was accompanied with an extreme upregulation of ICAM-1. Thus, it is interesting that the transported polyphenols from RDE were able to prevent different mechanisms mediating inflammatory responses triggered in EA.hy926 cells, implying a potential broad-spectrum anti-inflammatory property of transported polyphenols from RDE. As mentioned above, polyphenols (quercetin-3-glucoside, quercetin-glucuronide, rutin, quercetin-3-O-malonylglucoside, and kaempferol-glucoside) in RDE were absorbed and transported across the Caco-2 BBe1 cell monolayer, and thus were directly in contact with the EA.hy926 cells located on the basolateral compartment. Previous studies have revealed the inhibitory effects of polyphenols from different natural sources on the production of TNF-α, IL-8, ICAM-1, and VCAM-1 in TNF-α inflamed endothelial cells [36,37]. Similarly, the anti-inflammatory effects of RDE observed in this study can be attributed to the transported quercetin glycoside derivatives by the Caco-2 BBe1 cells. 

Our recent study has applied the co-culture model of Caco-2/ EA.hy926 to investigate the peptides uptake and involved mechanisms [38]. Although monolayer Caco-2 cells in the co-culture model may mimic the gut-blood-barrier [39], it has some limitations for investigating the transport dynamics of the intestine due to a deficient expression of brush border. The Caco-2 BBe1 (brush border expressing), a cloned cell line from the colon, can express the brush border under an in vitro condition. We optimized the co-culture model by using Caco-2 BBe1 cells instead of Caco-2 cells, which might shrink the gap of transport dynamics between in vitro co-culture model and in vivo condition. The present study was the first one using the optimized model of Caco-2 BBe1/ EA.hy926 to mimic the in vivo absorption and transportation of polyphenols in the RDE. This setup provides an excellent example of the transport model in which the potential absorption kinetics between the intestinal epithelial cell and vascular endothelial cell are considered.

## 5. Conclusions

In summary, this study clearly identified the species and concentrations of RDE-originated polyphenols that can be absorbed and transported by the Caco-2 BBe1 monolayer. It was also the first study to demonstrate that polyphenols of RDE, particularly the transported quercetin glycosides (quercetin-3-glucoside, quercetin-glucuronide, rutin, quercetin-3-*O*-malonylglucoside, and kaempferol-glucoside) can effectively suppress inflammatory responses in the co-culture model of intestinal epithelial cells and vascular endothelial cells. The results suggest that RDE might be useful as a natural resource of polyphenols for preventing intestinal malfunction and CVDs associated with chronic inflammation. Although current findings are provoking, more in vivo studies are required to validate the preventive effects of RDE on CVDs. 

## Figures and Tables

**Table 1:** Primers used in this study.

Gene	Primer Sequences (5′→3′)	Length (bp)	Access No.
IL-8	AGTCCTTGTTCCACTGTGCC	104	NM_000584.4
	GTGCTTCCACATGTCCTCAC		
TNF-α	CATTGCCCTGTGAGGAGGAC	131	NM_000594.4
	CGACCCTAAGCCCCCAATTC		
IL-6	CCAGCTATGAACTCCTTCTC	234	NM_001318095.1
	GCTTGTTCCTCACATCTCTC		
ICAM-1	TCATCACTGTGGTAGCAGCC	159	NM_000201.3
	GATAGGTTCAGGGAGGCGTG		
VCAM-1	AATTCCACGCTGACCCTGAG	151	NM_001078.4
	GGCCACCACTCATCTCGATT		
COX-2	GAATGGGGTGATGAGCAGTT	561	NM_000963.4
	CAGAAGGGCAGGATACAGC		
IL-10	TGGAATGCGAGCAATCCTGA	148	NM_000572.3
	TTACCTGGAGGAGGTGATGC		
	GGCCTTGCTCTTGTTTTCAC		
GAPDH	GCACCGTCAAGGCTGAGAAC	142	NM_001289745.2
	ATGGTGGTGAAGACGCCAGT		

Notes: interleukin-8 (IL-8), intercellular adhesion molecule-1 (ICAM-1), tumor necrosis factor-alpha (TNF-α), interleukin-6 (IL-6), interleukin-10 (IL-10), cyclooxygenase 2 (COX-2), vascular cell adhesion molecule 1 (VCAM-1), glyceraldehyde-3-phosphate dehydrogenase (GAPDH), base pair (bp).

## Abbreviations

RDE	red-osier dogwood extract
CVD	cardiovascular disease
IL-8	interleukin-8
TNF-α	tumor necrosis factor-alph
IL-6	interleukin-6
ICAM-1	intercellular adhesion molecule-1
VCAM-1	vascular cell adhesion molecule 1
COX-2	cyclooxygenase 2
ox-LDL	oxidized low-density lipoprotein
HBSS	Hank’s balanced salt solution
FBS	fetal bovine serum
TEER	transepithelial electrical resistance
ELISA	enzyme-linked immunosorbent assay
real-time PCR	real-time polymerase chain reaction
FA	formic acid
SPE	solid phase extraction
VEGF	vascular endothelial growth factor
